# Prolyl-isomerase Pin1 controls Notch3 protein expression and regulates T-ALL progression

**DOI:** 10.1038/onc.2016.5

**Published:** 2016-02-15

**Authors:** G Franciosa, G Diluvio, F Del Gaudio, M V Giuli, R Palermo, P Grazioli, A F Campese, C Talora, D Bellavia, G D'Amati, Z M Besharat, C Nicoletti, C W Siebel, L Choy, A Rustighi, G Del Sal, I Screpanti, S Checquolo

**Affiliations:** 1 Laboratory of Molecular Pathology, Department of Molecular Medicine, Sapienza University, Rome, Italy; 2 Center for Life Nano Science@Sapienza, Istituto Italiano di Tecnologia, Rome, Italy; 3 Department of Radiological, Oncological and Pathological Sciences, Sapienza University, Rome, Italy; 4 Unit of Histology and Medical Embryology, Department of Anatomy, Histology, Forensic Medicine and Orthopaedics, Sapienza University, Rome, Italy; 5 Department of Discovery Oncology, Genentech, South San Francisco, CA, USA; 6 Laboratorio Nazionale CIB Area Science Park Trieste, University of Trieste, Trieste, Italy; 7 Department Life Sciences, University of Trieste, Trieste, Italy; 8 Institute Pasteur-Foundation Cenci Bolognetti, Sapienza University, Rome, Italy; 9 Department of Medico-Surgical Sciences and Biotechnology, Sapienza University, Latina, Italy

## Abstract

Deregulated Notch signaling is associated with T-cell Acute Lymphoblastic Leukemia (T-ALL) development and progression. Increasing evidence reveals that Notch pathway has an important role in the invasion ability of tumor cells, including leukemia, although the underlying molecular mechanisms remain mostly unclear. Here, we show that Notch3 is a novel target protein of the prolyl-isomerase Pin1, which is able to regulate Notch3 protein processing and to stabilize the cleaved product, leading to the increased expression of the intracellular domain (N3_IC_), finally enhancing Notch3-dependent invasiveness properties. We demonstrate that the combined inhibition of Notch3 and Pin1 in the Notch3-overexpressing human leukemic TALL-1 cells reduces their high invasive potential, by decreasing the expression of the matrix metalloprotease MMP9. Consistently, Pin1 depletion in a mouse model of Notch3-induced T-ALL, by reducing N3_IC_ expression and signaling, impairs the expansion/invasiveness of CD4^+^CD8^+^ DP cells in peripheral lymphoid and non-lymphoid organs. Notably, in *in silico* gene expression analysis of human T-ALL samples we observed a significant correlation between Pin1 and Notch3 expression levels, which may further suggest a key role of the newly identified Notch3-Pin1 axis in T-ALL aggressiveness and progression. Thus, combined suppression of Pin1 and Notch3 proteins may be exploited as an additional target therapy for T-ALL.

## Introduction

Notch pathway is an evolutionarily conserved signaling pathway, which has an important role in cell-fate determination and differentiation in many tissues.^1^ Aberrant Notch signaling has been involved in the pathogenesis of human diseases,^2^ including T-cell acute lymphoblastic leukemias (T-ALLs), which represents approximately 15 and 25% of ALLs seen in children and adults, respectively.^3, 4^ Constitutive activation of either Notch1 or Notch3 is able to induce efficiently T-ALL in mouse models, closely resembling their human counterparts.^5, 6, 7, 8^ Activating mutations in Notch1 have been identified in over 60% of human T-ALL,^9, 10^ whereas Notch3 overexpression has been shown in most human T-ALL samples.^8, 11^ The absence of Notch3 genetic modifications in T-ALL implies that other mechanisms such as transcriptional, epigenetic, post-translational or a combination of these are responsible for its overexpression. Altered degradation process and/or acetylation/deacetylation balance have been shown to have an important role in the control of Notch3 protein stability,^12, 13^ thus contributing to the sustained Notch3 overexpression and Notch3-dependent leukemia development in Notch3 transgenic mice.^7^ These observations suggest that Notch3 expression can be modified by more than one type of post-translational modification (PTM) event.^14^

Increasing evidence reveals a key role of PTMs in the initiation, development and progression of several diseases, including cancer.^10^ Reversible phosphorylation, that is, addition of a phosphate group to the serine, threonine and tyrosine residues is a ubiquitous regulatory mechanism and was one of the first PTMs to be described. The peptidyl-prolyl Pin1 *cis/trans* isomerase was discovered as an enzyme that specifically recognizes and binds to phosphorylated Serines or Threonines preceding a Proline (phospho Ser/Thr-Pro) residue inducing conformational changes of phospho-proteins.^15^ Pin1 is a unique prolyl-isomerase that transduces phosphorylation signaling by affecting the functions of its substrates, including protein stability, catalytic activity, phosphorylation status, protein–protein interactions and/or subcellular localization.^15, 16, 17^ Pin1 alterations have been implicated in the amplification of oncogenic signals, by stabilizing oncoproteins and/or destabilizing or inactivating tumor suppressors,^15, 18^ as also shown by its frequent deregulation in several human malignancies.^16^ Moreover, recent studies suggested a pivotal role of Pin1 in increasing the oncogenic activity of Notch1 protein in breast cancer development and progression.^19, 20^ However, whether Pin1 might directly act on Notch expression and/or function in leukemias is not known. To this end, we evaluated the possible crosstalk between Pin1 and Notch proteins in T-ALL context, by analyzing human T-ALL cell lines and a mouse model of Notch3-induced T-ALL.^7^ Here, we show that Notch3 is a novel target of Pin1 isomerase. The Notch3-Pin1 binding regulates Notch3 protein expression and signaling, through a dual mechanism that impinges on its cleavage at the cell membrane and on the stability of its released intracellular domain. Notably, Pin1 deletion in N3IC-tg mice prevents the acquisition of an invasive malignant phenotype of T-ALL.

Together, our findings demonstrate that Pin1–Notch3 axis may reinforce Notch signaling effect in T-ALL, by influencing tumor grade and aggressiveness, finally suggesting that their combined inhibition may be exploited in target therapy protocols.

## Results

### Pin1 regulates Notch3 expression in T-ALL cell lines

**Figure 1:**
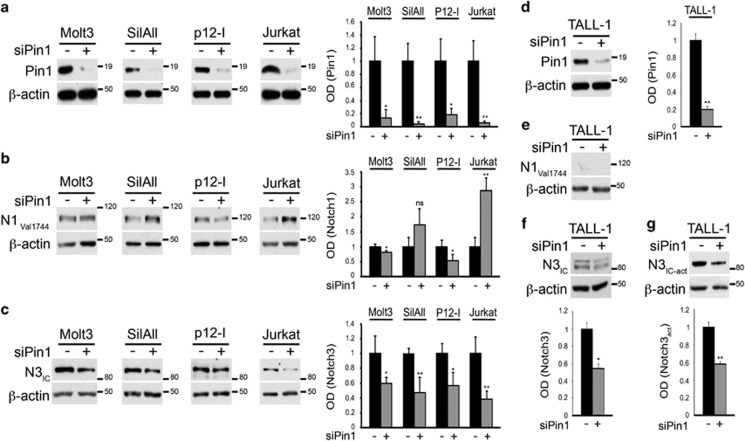
Pin1 silencing modulates the Notch3 protein expression in human T-ALL cell lines. Activated Notch1 (Notch1_Val1744_) and Notch3 (N3_IC_) expression in response to Pin1 silencing in (**b**, **c**) Notch1-activated (Molt3, SilAll, P12-Ichikawa and Jurkat) and (**e**–**g**) Notch1-non activated/Notch3 activated (N3_IC-act_) overexpressing (TALL-1) human T-ALL cell lines. (**a**, **d**) Western blots against Pin1 show the efficiency of Pin1 silencing (siPin1) (left panels). Western blot against the anti-β-actin was used as a loading control. All the western blots in the figure are representative of at least three independent experiments, each in triplicate. In all right (**a**–**d**) and lower (**f**, **g**) panels are shown the optical densitometry (OD) of Pin1 (**a**, **d**), Notch1 (**b**) and Notch3 (**c**, **f**, **g**) protein expression levels analyzed in all the experiments performed, thus including the *P*-values, calculated using Student's *T*-test (i.e., ns, not significant *P>*0.05; *P⩽0.05; **P⩽0.01).

To analyze the putative role of Pin1 isomerase on both Notch1 and Notch3 protein expression and function in T-ALL context, Pin1 expression was silenced in different human T-ALL cell lines (Molt3, SilAll, P12-Ichikawa and Jurkat), all constitutively expressing activated Notch1-IC (N1_Val1744_) and Notch3-IC (N3_IC_) as shown in Figures 1b and c, respectively. The efficiency of Pin1 silencing was evaluated by western blot of Pin1 (Figure 1a). In the absence of Pin1, the levels of activated Notch1-IC are variably affected, appearing increased in SilAll and Jurkat cells, whereas decreased in Molt3 and P12-Ichikawa cells (Figure 1b), highlighting the lack of correlation between high Pin1 levels and the upregulation of Notch1-IC protein levels in human T-ALL cells, as instead previously described in breast cancer.^19, 20^ Notably, the levels of N3_IC_ decreased in all the cell lines analyzed, independently of Notch1 activation status, as revealed by the immunoreactivity to the anti-Notch1_Val1744_ antibody (Figure 1c). This is also evident in Pin1-silenced TALL-1 cells (Figures 1d, f and g), which neither bear Notch1-activating mutations nor display Notch1 activation^21^ and Figure 1e), while displaying constitutive activation of Notch3,^22^ also confirmed by the immunoreactivity to the specific anti-N3_IC-act_ antibody (Figure 1g). Taken together, these results suggest that Pin1 knockdown may have a specific role upon Notch3 protein in T-ALL context. Furthermore, we performed an *in silico* analysis of the Notch3 and Pin1 gene expression levels in several T-ALL cell lines^23, 24, 25^ (Supplementary Figure 1a and Supplementary Table S1) and in a cohort of 117 T-ALL pediatric patients^26^ (Supplementary Figure 1b). The analysis highlighted a significant direct correlation between Pin1 and Notch3 gene expression levels, allowing us to hypothesize a possible direct relationship between Pin1 and Notch3 at the protein level in T-ALL context.

### Combined inhibition of Pin1 and Notch3 results in a reduced T-ALL invasiveness

**Figure 2:**
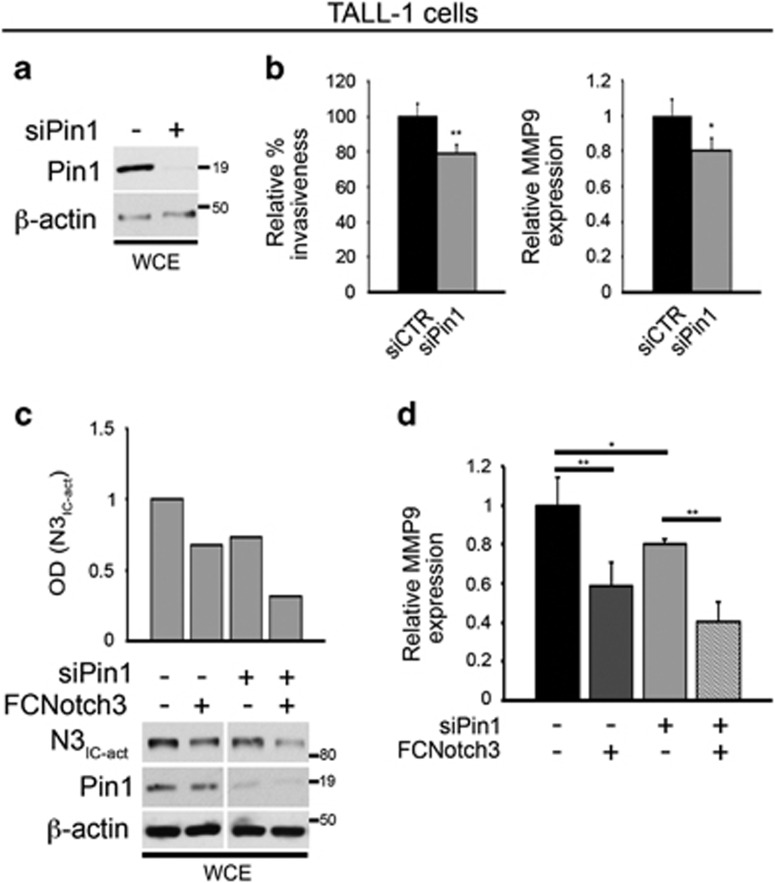
Pin1 silencing influences the TALL-1 cells invasiveness by regulating N3_IC_ protein expression. (**a**) Western blots against Pin1 show the efficiency of Pin1 silencing in TALL-1 cell line (siPin1). (**b**) TALL-1 cell line silenced or not for Pin1 was used in invasion Matrigel assay: relative percentage of invasiveness is shown with respect to the negative control, siCTR (left panel). RT–PCRs show downmodulation of MMP9 mRNA expression in Pin1-silenced cells (siPin1) with respect to the control cells (siCTR) (right panel). (**c**) Western blots against activated-N3_IC_ protein (N3_IC-act_) and Pin1 show the efficiency of the Notch3 receptor block and Pin1 silencing, respectively (lower panels). Optical densitometry (OD) of the activated-N3_IC_ protein expression (upper panel). (**d**) RT–PCRs show downmodulation of MMP9 mRNA expression in Notch3-blocked Pin1-silenced cells (siPin1+FCNotch3) with respect to both Notch3-blocked or Pin1-silenced controls alone. In both panels (**a**) and (**c**), western blot against the anti-β-actin was used as a loading control. All the results shown in the figure are expressed as the means average deviations of three separate experiments, each in triplicate, and *P*-values were calculated using Student's *T*-test (i.e., ns, not significant *P>*0.05; *P⩽0.05; **P⩽0.01). WCEs, whole-cell extracts.

Digging deeper into the effects of Pin1 depletion in leukemic cells, we focused our *in vitro* study on the Notch3-overexpressing TALL-1 leukemic cells,^22^ described above. We first evaluated whether the absence of Pin1 could affect cell growth or viability in such cells (Figure 2a and Supplementary Figure 2a). Despite the decreased N3_IC_ expression levels previously observed (Figures 1f and g), Pin1 seems not to be required for TALL-1 cell growth (Supplementary Figure 2a). Next, we focused on TALL-1 invasive properties, since it has been demonstrated that high levels of Pin1 correlate with high tumor grade and aggressiveness in breast cancer.^27^ By using matrigel-coated invasion chambers to simulate extracellular matrix, known to be degraded during tumor dissemination,^28^ we observed a significant decrease in the invasion of Pin1-silenced TALL-1 cells when compared with the control cells (Figure 2b, left panel). Notably, this reduction correlates with the decrease in the pro-invasive matrix metalloproteinase MMP9 expression levels (Figure 2b, right panel), known to be involved in the extracellular matrix degradation, thus promoting tumor progression.^29, 30^ Furthermore, as shown in Figure 2c, when Pin1 silencing was combined with Notch3 blocking, we observed a significantly higher decrease in the activated-N3_IC_ protein levels with respect to control cells, or Pin1 or Notch3 singly inhibited (Figure 2c). Notably, the combined inhibition further reduced the MMP9 mRNA expression (Figure 2d), thus supporting the notion that Notch3 and Pin1 synergistically contribute to invasive properties of leukemic cells, without influencing their viability (Supplementary Figure 2b).

### Deletion of Pin1 correlates with the significant decrease in Notch3 protein expression and function *in vivo*

**Figure 3:**
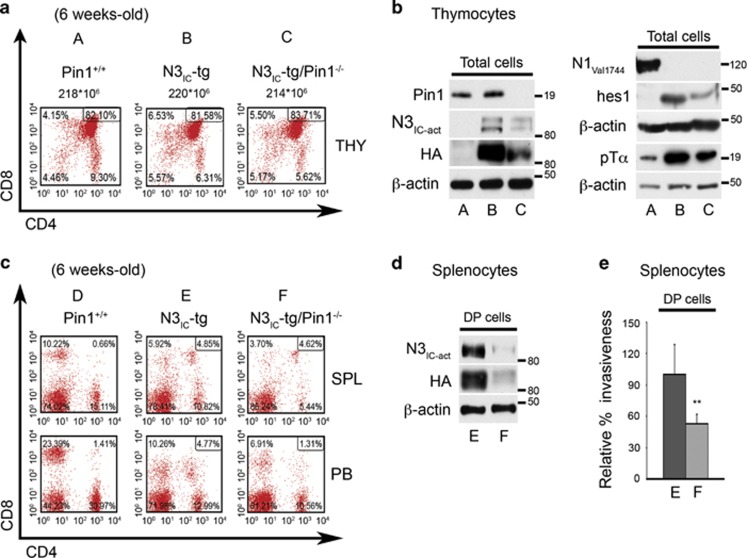
Pin1 ablation impairs Notch3 signaling in thymocytes of young N3_IC_ transgenic mice resulting in the decrease of expansion/invasiveness of CD4^+^CD8^+^ DP splenic cells. CD4^+^ and/or CD8^+^ subset distribution of thymocytes from representative 6-week-old Pin1^+/+^ (A), N3_IC_-tg (B) and N3_IC_-tg/Pin1^−/−^ (C) mice. (**b**) Whole-cell extracts from thymocytes illustrated in (**a**) were revealed with anti-Pin1, anti-activated N3_IC_ (N3_IC-act_), anti-HA (left panels) and anti-activated Notch1 (Notch1_Val1744_), anti-Hes1 and anti-pTα (right panels) antibodies. Western blot against the anti-β-actin was used as a loading control. (**c**) CD4^+^ and/or CD8^+^ subset distribution of lymphocytes derived from SPL and blood of representative 6-week-old Pin1^+/+^ (D), N3_IC_-tg (E) and N3_IC_-tg/Pin1^−/−^ (F) mice. (**d**) Sorted CD4^+^CD8^+^ (DP) splenocytes illustrated in (**c**) (circle around the number) were used for western blot analysis against anti-activated N3_IC_ (N3_IC-act_), anti-HA and anti-β-actin antibodies and (**e**) in invasion Matrigel assay: relative percentage of DP cells invasiveness from N3_IC_-tg/Pin1^−/−^ mice is shown with respect to N3_IC_-tg cells. Results are shown as the means average deviations of five independent experiments (*n=*3–5 mice per group) and *P*-values were calculated using Student's *T*-test (i.e., **P⩽0.01). In all panels described in (**a**, **c**), numbers inside each cytogram indicate the percentages of the corresponding subsets and the results are representative of five independent experiments (*n=*3–5 mice per group: Pin1^+/+^ (*n=*15), N3IC-tg (*n=*25) and N3IC-tg/Pin1^−/−^ mice (*n=*15)). THY, thymus. SPL, Spleen; PB, Peripheral Blood.

To deepen the study of the relationship between Pin1 and Notch3 *in vivo*, we utilized the Notch3-IC transgenic mice (N3_IC_-tg), a mouse model of Notch3-dependent T-ALL we previously generated,^7^ which overexpress constitutively activated Notch3-IC protein and lack the expression of the activated Notch1 protein (Figure 3b and Pelullo *et al.*^31^), thus resembling the leukemic human cell line TALL-1 (Figures 1e–g).^21, 22^ We generated double mutant mice (N3_IC_-tg/Pin1^−/−^) by intercrossing the N3_IC_-tg mice with the Pin1 knockout mice (Pin1^−/−^).^32^

Figure 3a shows that in N3_IC_-tg/Pin1^−/−^ and N3_IC_-tg young mice (6 weeks) the thymocyte subset distribution is similar with respect to CD4 and/or CD8 expression. However, the Pin1 ablation in N3_IC_-tg mice, confirmed by the absence of the Pin1 protein expression in double mutant thymocytes (Figure 3b, left panel), caused a significant reduction in the Notch3-IC protein expression levels in whole thymocyte extracts derived from N3_IC_-tg/Pin1^−/−^ mice when compared with N3_IC_-tg littermates (Figure 3b, left panel), as revealed by the western blot with either anti-N3_IC-act_ or anti-HA antibodies, the latter recognizing the HA-tagged N3IC transgene. Consistently, we also observed decreased protein expression levels of known Notch target genes, such as Hes1^33^ and pTalpha,^34^ when compared with N3_IC_-tg thymocytes (Figure 3b, right panel). Notably, neither Notch3-IC tg nor double mutant N3_IC_-tg/Pin1^−/−^ mice display activated Notch1 protein (Figure 3b, right panel).

We and others have previously shown that accumulation of CD4^+^CD8^+^ DP cells in spleen (SPL), lymph nodes and peripheral blood (PB) represents a pathognomonic feature of T-cell leukemias sustained by enforced expression of N_IC_ in pre-T-cells or in bone marrow of mice.^5, 7^ As shown in Figure 3c, upper panels, and Supplementary Figure 3a, DP cells appeared in the SPL of 6-week-old double mutant N3_IC_-tg/Pin1^−/−^ mice in a similarly increased percentage with respect to N3_IC_-tg littermates, when compared with wt (Pin1^+/+^) mice. On the contrary, in circulating blood of double mutant mice the percentage of DP cells was similar to that observed in wt mice, while being increased in N3_IC_-tg littermates (Figure 3c, lower panels, and Supplementary Figure 3b). Notably, similarly to what observed in thymocytes, Pin1-deleted DP transgenic splenocytes revealed a strong decrease in N3_IC_ protein levels, as revealed by the western blot performed on DP sorted cells (Figure 3d). These results, together with the *in vitro* results described above (Figure 2), suggest the possibility that Pin1 deletion in N3_IC_-tg mice, by decreasing N3_IC_ expression and signaling, could inhibit Notch3-IC-dependent tumor progression. In keeping with this, ablation of endogenous Pin1 significantly reduced DP sorted splenocytes cell invasiveness, measured in Matrigel invasion assay (Figure 3e), while their apoptotic or proliferative rate did not change (data not shown).

### Deletion of Pin1 prevents T-ALL progression in Notch3-IC tg mice

**Figure 4:**
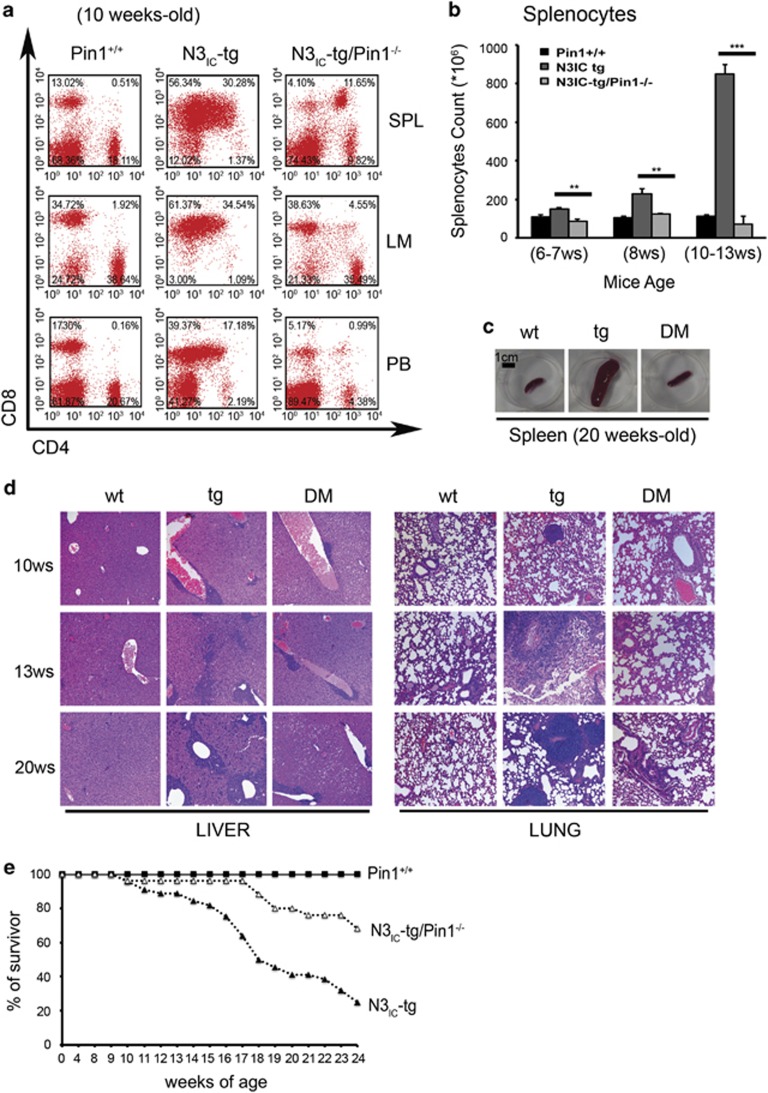
Pin1-induced N3_IC_ downregulation prevents T-ALL development and progression in N3_IC_ transgenic mice. (**a**) CD4^+^ and/or CD8^+^ subset distribution of lymphocytes derived from SPL, PB and mesenteric lymph nodes (LM) of representative 10-week-old Pin1^+/+^, N3_IC_-tg and N3_IC_-tg/Pin1^−/−^ mice. In all panels, numbers inside each cytogram indicate the percentages of the corresponding subsets and the results are representative of three independent experiments (*n=*3–5 mice/group: Pin1^+/+^ (*n=*9), N3IC-tg (*n=*15) and N3IC-tg/Pin1^−/−^ mice (*n=*12)). (**b**) Splenocytes count from Pin1^+/+^, N3_IC_-tg and N3_IC_-tg/Pin1^−/−^ mice at the age indicated. Values represent the means average deviations of 3–5 mice for each genetic background and *P*-values were calculated using Student's *T*-test (i.e., **P⩽0.01; ***P⩽0.001). (**c**) Macroscopic aspect of SPL isolated from 20-week-old Pin1^+/+^ (wt), N3_IC_-tg (tg) and double mutant N3_IC_-tg/Pin1^−/−^ (DM) mice. (**d**) Histological analysis of non-lymphoid organs (liver, upper and lung, down) from representative Pin1^+/+^ (wt), N3_IC_-tg (tg) and N3_IC_-tg/Pin1^−/−^ (DM) mice at the age indicated. Hematoxylin and Eosin staining, original magnification × 10. (**e**) Mortality curve of Pin1^+/+^, N3_IC_-tg and N3_IC_-tg/Pin1^−/−^ mice. The numbers of spontaneously dead mice were plotted against their age. Results are indicated as the percentage of surviving mice at each age. The follow-up of mice was stopped at 24 weeks, being 75% of the N3_IC_-tg mice dead at this age and 70% of N3_IC_-tg/Pin1^−/−^ mice survivor (*n=*50 for Pin1^+/+^; *n=*60 for N3_IC_-tg *n=*30 for N3_IC_-tg/Pin1^−/−^ mice).

To better address the role of Pin1–Notch3 axis in sustaining lymphoma cell migration/invasion and tumor progression, we analyzed the lymphoma development by evaluating the total cell yield and immunophenotype of SPL, lymph nodes and blood of double mutant N3_IC_-tg/Pin1^−/−^ mice with respect to N3_IC_-tg littermates (Figure 4a). The absence of DP cells in SPL, mesenteric lymph nodes and PB of wt mice represents the physiological condition in the absence of tumor (Figure 4a, left panels, and Supplementary Figures 3c–e). The analysis of different peripheral lymphoid tissues obtained from 10-week-old double mutant N3_IC_-tg/Pin1^−/−^ mice, age at which a massive presence of DP cells is usual in N3_IC_-tg mice (Figure 4a, middle panels, and Supplementary Figures 3c–e), showed the presence of a significantly reduced percentage of DP cells in SPL and lymph nodes (11.65 and 4.55% vs 30, 28 and 34.54%, respectively) (Figure 4a, right and middle panels, and Supplementary Figures 3c and d). Moreover, DP cells are almost absent (0.99%) in circulating blood of double mutant mice, while amounting to 17.18% in N3_IC_-tg littermates (Figure 4a, right and middle panels, and Supplementary Figure 3e). More importantly, N3_IC_-tg/Pin1^−/−^ double mutant mice never show neither splenomegaly nor other overt macroscopic or histological abnormalities of peripheral lymphoid organs, like commonly observed in N3_IC_-tg littermates (Figures 4b and c and data not shown). Consistently, while the non-lymphoid organs (liver and lung) of N3_IC_-tg mice showed a massive and diffuse tumor cell infiltration (Figure 4d, middle panels), already evident at 10 weeks of age, the N3_IC_-tg/Pin1^−/−^ double mutant mice only display a little lymphoid cell infiltration around the vessels, restricted to the liver at late age (Figure 4d, right panels).

Moreover, such a huge difference in phenotype observed between N3IC-tg/Pin1^−/−^ mice with respect to N3_IC_-tg counterparts correlates with significant survival differences: indeed, while 75% of N3IC-tg mice died at 24 weeks of age, displaying phenotypic features of T-cell lymphoblastic lymphoma,^7^ 70% of N3IC-tg/Pin1^−/−^ mice were still alive at the same age (Figure 4e). Less than half of the N3IC-tg/Pin1^−/−^ mice that died spontaneously after the age of 18 weeks displayed tumor development at autopsy. Therefore, N3IC-tg/Pin1^−/−^ mice develop tumors, albeit with a reduced kinetics, only in less than 5% of cases. Overall, these data indicate that Pin1 deletion affects the Notch3-dependent tumor progression and invasion properties of lymphoma cells *in vivo*.

### Pin1 directly binds to Notch3 and influences its processing and stability in *ex vivo* and *in vivo* systems

**Figure 5:**
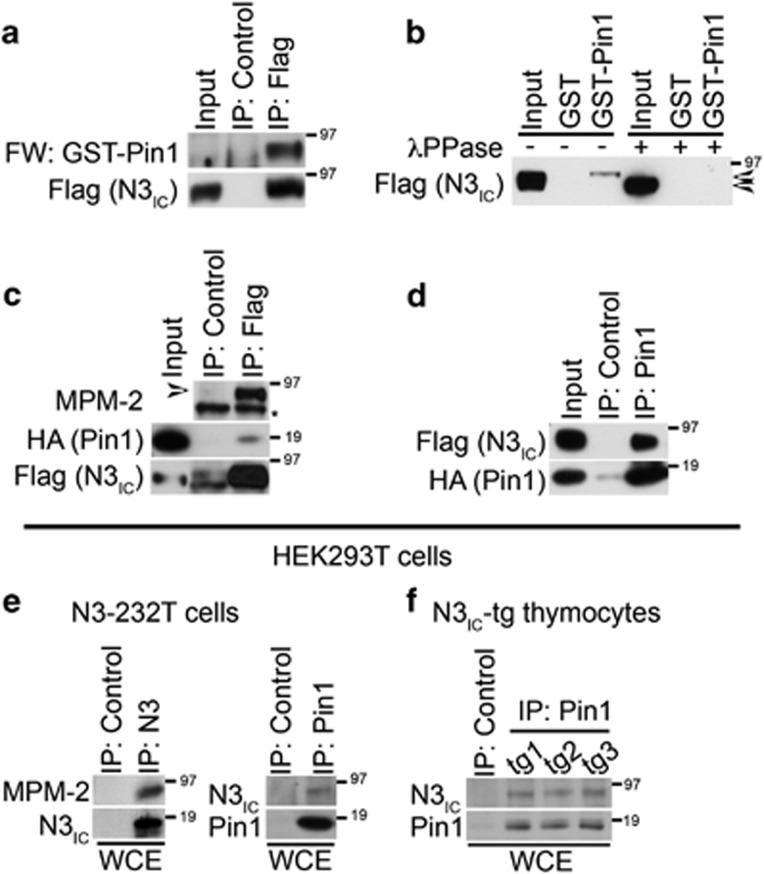
Pin1 directly interacts with Notch3. (**a**) Control or anti-Flag antibody immunoprecipitates from HEK293T cells transfected with Flag N3IC-wt were subjected to far western blotting using purified GST–Pin1 as a probe, followed by anti-Pin1 immunoblotting. Anti-Flag western blot analysis of the upper panel after stripping is shown. (**b**) Lysates used in (**a**), previous treated with lamba phosphatase (+), were subjected to GST or GST–Pin1 pulldown followed by anti-Flag western blotting. The arrows indicate the phosphorylated (upper band) and the non-phosphorylated (lower band) forms. (**c**) Control or anti-Flag antibody immunoprecipitates from HEK293T cells co-transfected with Flag N3IC-wt and HA-Pin1 plasmids were subjected to western blot and probes with anti-MPM-2, to detect the Notch3 phosphorylation levels at Ser/Thr-Pro sites, followed by stripping and anti-Flag western analysis to show N3_IC_ immunoprecipitated protein levels. The blot with anti-HA antibody was used to reveal the Notch3-Pin1 binding (middle panel). The * indicates a non-specific band. (**d**) Control or anti-Pin1 antibody immunoprecipitates from the same cells used in (**c**) were probes with anti-Flag, to detect the Notch3-Pin1 binding, and with the anti-HA antibody to show Pin1 immunoprecipitated protein levels. (**e**) Anti-Notch3 (left panel) and anti-Pin1 (right panel) immunoprecipitates from N3–232 T cells were subjected to western blot and probes with anti-MPM2 antibody, to detect the Notch3 phosphorylation levels at Ser/Thr-Pro sites, and anti-N3_IC_ antibody to detect endogenous Notch3–Pin1 interaction, respectively. In both panels (**e**), the blots with anti-N3_IC_ and anti-Pin1 antibodies were used to show Notch3 and Pin1 immunoprecipitated protein levels, respectively. (**f**) Anti-Pin1 immunoprecipitates from N3IC-tg thymocytes were subjected to western blot and probes with anti-N3_IC_ and anti-Pin1 antibodies, to detect endogenous Notch3–Pin1 interaction and Pin1 immunoprecipitated protein levels, respectively. The input lane indicated in all the western blot of (**a**–**d**) shows 5% of total lysate. All data are representative of at least three independent experiments, each in triplicate. WCEs, whole-cell extracts.

Pin1 isomerase is able to bind its substrates only through specific phosphorylated Ser/Thr-Pro motifs,^35^ recognized by a specific antibody (named MPM-2).^36^ Notch3 intracellular region (N3_IC_) harbors many of these Ser/Thr-Pro motifs, whose phosphorylation may generate Pin1-binding sites. Indeed, by analyzing the Pin1–Notch3 interaction *in vitro* using a recombinant GST–Pin1, we demonstrated that this interaction is direct (Figure 5a) and phosphorylation dependent, as treatment with phosphatases induces loss of binding (Figure 5b). Moreover, by using Flag-tagged Notch3-IC (Flag-N3_IC_) and HA-Pin1 plasmids in co-transfection experiments, we observed that N3_IC_ is indeed phosphorylated in Ser/Thr-Pro motifs (Figure 5c), as revealed by the immunoreactivity to MPM2, and both proteins reciprocally co-immunoprecipitated (Figures 5c and d). Notch3 and Pin1 interaction occurs also *in vivo*, as endogenous Notch3 expressed in N3-232 T murine leukemic T cells, we established from N3_IC_-tg mice,^7^ was recognized by the specific MPM-2 antibody (Figure 5e, left panel) and was able to bind to endogenous Pin1 protein (Figure 5e, right panel). Most importantly, endogenous Notch3 is able to bind Pin1 also in thymocytes freshly obtained from N3_IC_-tg mice (Figure 5f).

**Figure 6:**
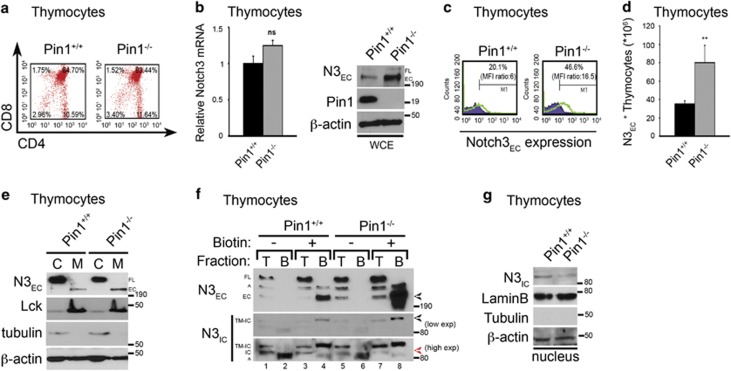
Pin1 affects Notch3 processing. (**a**) CD4^+^ and/or CD8^+^ subset distribution of thymocytes from Pin1^+/+^ and Pin1^−/−^ mice. In both panels, numbers inside each cytogram indicate the percentages of the corresponding subsets. (**b**) RT–PCR shows the unchanged relative Notch3 mRNA levels in Pin1^−/−^ vs Pin1^+/+^ thymocytes (left panel). (Right panel) Western blot analysis of whole-cell extracts (WCEs) from the same thymocytes probed with anti-Notch3EC (N3_EC_) and anti-Pin1 antibodies. The β-actin expression was used as a loading control. (**c**) Notch3 extracellular expression (N3_EC_) from thymocytes of Pin1^+/+^ and Pin1^−/−^ mice indicated as percentages inside each cytogram. The violet curve represents the isotypic control. The mean fluorescence intensity (MFI) ratio between Notch3 and isotypic control staining is also indicated. The results showed in both panels are representative of five independent experiments (*n=*5 mice for group). (**d**) Bar graphs represent the absolute cell number from thymocytes expressing N3_EC_ of the same mice indicated in (**c**). (**e**) Cytosolic (C) and membrane (M) fractions from Pin1^+/+^ and Pin1^−/−^ thymocytes were analyzed in immunoblot assays to detect the N3_EC_ expression. Anti-Lck and anti-α-tubulin were used as fraction markers; anti-β-actin was used as a loading control. (**f**) Thymocytes from Pin1^+/+^ and Pin1^−/−^ mice were incubated with EZ-Link Sulfo-NHS-SS-Biotin (+) or were mock (−) treated, as described in Materials and methods. Cells were lysed and extracts were loaded on a 6% SDS–PAGE gel either directly (T fraction, 15% of the extract) or after incubation on streptavidin-agarose beads (B fraction, 85% of the extract). Extracts were then immunoblotted with the anti-N3_EC_ and anti-N3_IC_ antibodies. Positions of the 210-kDa Notch3 extracellular (EC) and 97-kDa Notch3 transmembrane-intracellular (TM-IC) domains are indicated by black arrows. In the high exposition is indicated the position of the Notch3 intracellular domain (IC) (red arrow). ^ indicates non-specific bands. (**g**) Nuclear fractions from Pin1^+/+^ and Pin1^−/−^ thymocytes were analyzed in immunoblot assays to detect the N3_IC_ expression. Anti-LaminB and anti-α-tubulin were used as fraction markers; anti-β-actin was used as a loading control. In all panels (**b**) and (**d**), results are shown as the means average deviations of five separate experiments and *P*-values were calculated using Student's *T*-test (i.e., ns, not significant *P>*0.05; **P⩽0.01). In all the western blots represented in the figure, FL indicates Notch3 full-length receptor and EC indicates extracellular region.

To investigate in detail the molecular mechanism by which Pin1 regulates Notch3 expression and/or function, we first analyzed the endogenous Notch3 mRNA and protein expression levels in thymocytes derived from wild-type (Pin1^+/+^) and Pin1 knockout (Pin1^−/−^) mice,^32^ which show similar thymocyte subset distribution with respect to CD4 and/or CD8 expression (Figure 6a). As shown in Figure 6b, despite the absence of significant difference of Notch3 mRNA level (left panel), western blot analysis revealed a strong increase in Notch3 protein expression in Pin1^−/−^ thymocytes (right panel) with respect to wt ones, as indicated by its extracellular fragment expression (N3_EC_). To validate this observation, we further analyzed the Notch3 receptor expression at the surface of the same cells by using different techniques, that is, FACS analysis (Figures 6c and d), western blot of membrane/cytosol fractions (Figure 6e and Supplementary Figure 4a) and biotinylation assay (Figure 6f and Supplementary Figure 4b). As shown in Figure 6c, Pin1^−/−^ thymocytes display a significant increase in the N3_EC_ surface expression, either when the percent distribution/mean fluorescence intensity (Figure 6c) or the absolute number is considered (Figure 6d). Consistently, this occurs also in the membrane fractions of the Pin1^−/−^ thymocytes when compared with those from Pin1^+/+^ littermates (Figure 6e and Supplementary Figure 4a). The biotinylation assay shown in Figure 6f further confirms the strong accumulation of Notch3 receptor at the cell surface of Pin1-deleted thymocytes (compare lanes 4 and 8), in both 220-kDa (N3_EC_) and 97-kDa (TM-IC) Notch3 cleavage products. Since we found that the relative amounts of Notch3 mRNA were essentially identical in both mice (Figure 6b), we established that this accumulation could not be due to an increased production of Notch3 but more likely to an impaired clearance of the Notch3 receptor from the cell surface, probably due to a defect in its processing. Indeed, N3_IC_ levels decreased in Pin1^−/−^ total thymocytes of both un-biotinylated (compare lanes 1 and 5) and biotinylated (compare lanes 3 and 7) fractions with respect to Pin1^+/+^ thymocytes counterpart (Figure 6f, lower panel, blot: N3_IC_ high exposition and Supplementary Figure 4b). Consistently, Notch3 intracellular domain (N3_IC_) was not detected in the nucleus of Pin1^−/−^ thymocytes (Figure 6g).

Overall, our data indicate that Pin1 is able to affect the levels of endogenous Notch3 possibly by influencing its receptor processing at the cell surface. In keeping with these observations, the Pin1 ablation in N3_IC_-tg mice caused a significant increase in surface Notch3 receptor per cell (Supplementary Figure 5), as highlighted by the increase in the N3_EC_ mean fluorescence intensity observed in both DP thymocytes and DP splenocytes derived from young N3_IC_-tg/Pin1^−/−^ double mutant mice described in Figure 3, with respect to the N3_IC_-tg littermates.

**Figure 7:**
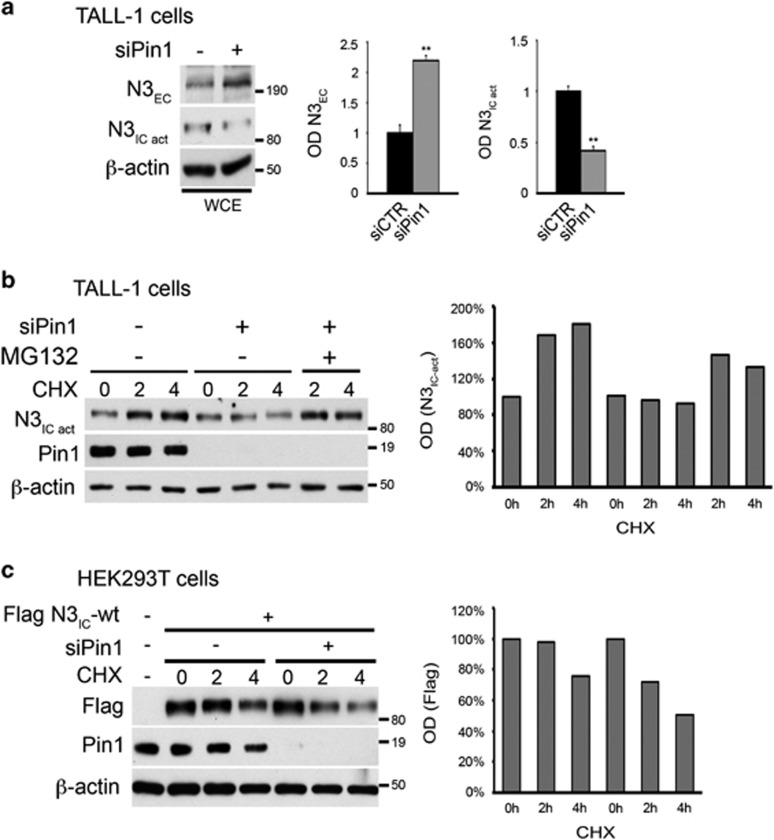
Pin1 influences Notch3 processing and stability in endogenous and exogenous system. (**a**) Western blot analysis of Notch3 extracellular (N3_EC_) and activated intracellular (N3_IC-act_) protein expression of whole-cell extract (WCE) derived from Pin1-silenced TALL-1 (+) vs control cells (−) (left panel). The western blots in the figure are representative of at least three independent experiments, each in triplicate. The optical densitometry (OD) (right panels) was analyzed in all the experiments performed, thus including the *P*-values, calculated using Student's *T*-test (i.e., **P⩽0.01). (**b**) WCEs from Pin1-silenced TALL-1 cells (+) vs control cells (−) in a time course assay with 10 μg/ml of cycloheximide (CHX), in the presence or absence of the proteasome inhibitor MG132 for the same times before lysis, were revealed by immunoblotting with anti-activated N3_IC_ (N3_IC-act_), anti-Pin1 and anti-β-actin antibodies (left panel). The right panel shows the relative quantification of activated-N3_IC_ as determined by OD. (**c**) Left panel, Western blot analysis of whole-cell extracts from HEK293T cells transfected with Flag N3IC-wt plasmid and silenced for Pin1 (+) or control (−) in a time course assay with 10 μg/ml of cycloheximide (CHX). Extracts were immunoblotted with anti-Flag, anti-Pin1 and anti-β-actin antibodies. The right panel shows the relative quantification of Flag N3IC as determined by OD. All data are representative of at least three independent experiments, each in triplicate.

Consistent with these data, Pin1-silenced human TALL-1 cells showed a significant increase in N3_EC_ protein expression (Figure 7a), while activated-N3_IC_ strongly decreased (Figure 7a), as previously observed (Figure 1g). Intriguingly, we also observed a significant decrease in N3-HA transgene expression in N3_IC_-tg/Pin1^−/−^ double mutant mice (Figure 3b, left panel, and Figure 3d), which suggests a role of Pin1 also directly on N3_IC_ protein. Notably, it has been demonstrated that Pin1 is able to affect both Notch1 processing and stability in breast cancer context.^19, 20^ Therefore, we analyzed the endogenous N3_IC_ protein stability in TALL-1 cells after Pin1 silencing in the presence of the protein synthesis inhibitor, cycloheximide (CHX). As shown in Figure 7b, while the N3_IC_ protein expression increased in the presence of CHX, necessarily generated from its cell surface cleavage, the Pin1 silencing prevented this effect but seems not to influence directly the N3_IC_ half-life with respect to the control cells, thus confirming in this context the predominant Pin1 role on Notch3 receptor processing previously observed in *ex vivo* Pin1^−/−^ thymocytes (Figure 6). However, the addition of the proteasome inhibitor MG132 in Pin1-silenced cells is able to rescue the N3_IC_ decreased expression, suggesting the involvement of the proteasome degradation system in the regulation of N3_IC_ protein levels observed in the absence of Pin1. To value this possibility, avoiding the N3_IC_ generation from the cell surface, we used HEK293T cells where flag-N3IC plasmid was overexpressed and endogenous Pin1 was silenced: as shown in Figure 7c, the half-life of N3_IC_ was reduced in Pin1-silenced cells when compared with cells silenced with the control siRNA (Figure 7c). Overall, these data suggest that Pin1 could have a dual role on Notch3 in T-ALL context, sustaining the cleavage of Notch3-IC on one side and protecting it from degradation on the other side, finally resulting in the increased intracellular levels of Notch3_IC_ protein.

## Discussion

T-ALL patients have a higher percentage of induction failure, rate of relapse and invasion into the central nervous system, when compared with the majority of ALL patients. An important role of Notch signaling in the progression and aggressiveness of solid tumors has been demonstrated;^37, 38, 39, 40^ in addition, there is increasing evidence supporting the same important role also in leukemia.^41^ In particular, it has been shown that Notch1 activation in T-ALL cells contributes to central nervous system infiltration^42^ and more recently that Notch1 influences both hypoxia-induced invasion property^43^ and extra-medullary infiltration of T-ALLs.^44^

In this study, we demonstrated that Pin1, by influencing N3_IC_ expression and signaling, contributes to T-ALL cell invasiveness properties both *in vitro* and *in vivo*. Indeed, we report a significant decrease in the pro-invasive protease MMP9 levels when Notch3-overexpressing human TALL-1 leukemic cells are inhibited for both Notch3 and Pin1 protein expression, also confirming the known Notch-dependent regulation of MMP expression genes.^37, 38, 41^

We genetically demonstrated that Pin1 deletion in Notch3-IC transgenic mice blocks the expansion/invasiveness of CD4^+^CD8^+^ DP cells in peripheral lymphoid and non-lymphoid organs and in circulating blood, finally preventing the progression of the lymphoproliferative disease. These results, together with previously reported observations,^13, 45^ confirm that the presence of CD4^+^CD8^+^ DP cells in PB and peripheral lymphoid organs represents a characteristic feature of T-ALL and further sustains that loss of DP in the periphery may represent a marker of drug treatment efficiency.^46^

Several mechanisms regulating Notch3 expression have been recently reported: miRNA-206 has been shown to inhibit colon cancer cell proliferation and migration through direct Notch3 targeting,^47^ as well as mir-150 does to regulate T-cell development.^48^ Moreover, methylation status regulation has been suggested to be responsible for Notch3 overexpression in T-ALL and several solid tumors.^49^ Additionally, several reports have demonstrated that Notch-IC is subjected to multiple phosphorylations in different domains that modulate its transcriptional activity by regulating its stability or subcellular localization^14^ while little is known about PTMs of the Notch receptor and their role in regulating Notch activity.^50, 51^ In this study, we report for the first time that Notch3 protein is subjected to phosphorylation in Ser/Thr-Pro motifs and that this PTM has an effect on its interaction with other protein, such as Pin1. Notably, more than 50% of human T-ALL patient samples show activating Notch1 mutations^9, 52^ whereas overexpression of Notch3, irrespective of gross abnormalities in the Notch3 locus, is a common finding in human T-ALL,^8^ raising the possibility that in this context high Pin1 expression might contribute to sustain high levels and function of N3_IC_ protein, similarly to what happens for Notch1 in breast cancer where it is rarely mutated.^20, 53^ Mechanistically, we show that Pin1 interacts directly with phosphorylated Notch3 and increases N3_IC_ protein expression. Furthermore, we observed that both Pin1-depleted thymocytes and splenocytes derived from Pin1^−/−^ and/or double mutant N3_IC_-tg/Pin1^−/−^ mice display a significant increase in the endogenous Notch3 extracellular expression at the cell surface, thus suggesting that the phosphorylation-dependent prolyl-isomerization catalyzed by Pin1 may regulate Notch3 receptor processing. More recently, an additional role of Pin1 on N1ICD stability has been shown in breast stem cells and in neural ischemic stroke, by protecting it from FBWX7 degradation.^20, 54^

We have recently demonstrated that the activation of Notch signaling decreases FBXW7 expression through the upregulation of mir-223 in T-ALL context,^55^ suggesting a positive loop in sustaining Notch-IC protein expression. Given the results reported in this paper, we can speculate that Pin1 could reinforce this loop, as it has been also demonstrated that Pin1 promotes FBWX7 self-ubiquitination and its proteasomal degradation by disrupting FBXW7 dimerization.^56^ Indeed, in addition to its role on Notch3 processing, in this study we also suggest a possible involvement of Pin1 in the control of Notch3_IC_ protein stability, which can justify the strong decrease in the exogenous Notch3-IC, recognized by the HA immunoreactivity, that we observed in both thymocytes and DP splenocytes of double mutant N3_IC_-tg/Pin1^−/−^ mice, which finally leads to the decreased levels of Notch3 target genes (pTalpha and Hes1). Taken together, these data support the hypothesis of a possible complex circuitry Notch3-Pin1-FBXW7 in T-ALL context, resulting in the regulation of Notch3_IC_ protein generation and stability, which remains to be investigated in more detail.

Overall, our findings provide new insights unveiling a possible dual mechanism focused on phosphorylation-dependent prolyl-isomerization by Pin1 as responsible for sustained Notch3-IC expression and signaling in T-cell leukemia. Notably, our data not only extend the role of Pin1 in regulating several members of Notch receptor family but underline the cell- (or tumor-) dependent context of Pin1 mechanistic activity. Indeed, here we show that in several human and mouse T-ALL models Pin1 specifically targets Notch3, without affecting Notch1 expression, thus suggesting a possible specific role on different Notch receptors, possibly also depending on different Pin1-target sites involved (data not shown).

Functionally, we show that the establishment of the Pin1/Notch3 relationship may contribute to promote Notch3-induced T-ALL aggressiveness, first by sustaining Notch3_IC_ protein levels. Notably, the influence of Pin1 on the Notch pathway is likely not limited to its direct action on Notch proteins. Indeed, several Notch targets are also Pin1 substrates (for example, cyclone D1 and NF-kB).^17^ This may suggest that Pin1 could promote T-ALL aggressiveness also by enhancing the activity of some Notch3-induced Pin1 targets, such as the NF-kB transcription factor, we previously showed to be activated by Notch3^57^ and known to be regulated by Pin1 isomerase also in leukemia/lymphoma development.^58^ In this way, Pin1 blocking may represent a new approach for targeting a common oncogenic mechanism to stop the multiple cancer-driving pathways, which act simultaneously in the disease. Indeed, more recently, it has been demonstrated that the Pin1 downmodulation by ATRA (all-*trans*-retinoic acid), known to be used in acute promyelocytic leukemia (APL) therapy,^59^ correlates with tumor cell growth inhibition in APL animal models and human APL cells *in vitro* as well as in APL patients.^60^ Notably, this occurs also in triple negative breast cancer, thus exerting a potent anticancer activity, probably by blocking Pin1 substrate oncogenes and tumor suppressors at the same time.^60^ In keeping with these findings, our work underlines for the first time that Pin1 isomerase could represent a new potential therapeutic target also in T-ALL treatment, by the regulation of Notch3 cancer-driving pathway. The significant direct correlation observed between Pin1 and Notch3 expression levels in human T-ALL cell lines and primary tumor samples confirms the possible relevance of our observations for human T-ALL development.

Our findings identify novel molecular mechanisms involved in the invasion of T-ALL cells. These results may provide a rationale for novel therapy approaches, as combined inhibition of Pin1 and Notch3 could suppress aggressive phenotypes, representing a useful tool to interfere with the mechanisms governing T-ALL cell extravasation into lymphoid and non-lymphoyd tissues, finally leading to the inhibition of migration and invasion processes.

## Materials and methods

### Mice

N3_IC_-tg/Pin1^−/−^ double mutant mice were generated by crossing N3_IC_-tg mice^7^ with Pin1^−/−^ mice.^32^ All mouse strains were on a C57BL/6 background. In all the experiments including mice studies at least nine animals for each genotype were used. The exact number of mice used in each experiment was reported in the relative figure legend. Different experimental groups were based on the age and genotype of the animals. No mice were excluded during the experiments. The studies involving animals have been conducted following the Italian National Guidelines for Animal Care, established in DL No. 26, 2014 and in accord with the Directive 2010/63/UE.

### Cell culture and treatments

HEK293T, murine N3-232T^7^ and human leukemic cells (Molt3, SilAll, p12-l, Jurkat and TALL-1)^55, 61^ were maintained as described elsewhere and all are mycoplasma-free. Cells were treated with 10 μM of GSI IX (DAPT) (Calbiochem, Darmstadt, Germany; Cat#565770) for 24 h. In some cases, TALL-1 cells were treated with: 30 μM proteasome inhibitor MG132 (Sigma, St Louis, MO, USA; Cat#C2211); 10 μg/ml ribosome inhibitor cycloheximide (Sigma; Cat#C4859) for the times indicated; 10 μg/ml of blocking anti-human Notch3 antibody (R&D Systems, Minneapolis, MN, USA; Cat#AF1559) for 48 h. Purified Sheep IgG (R&D Systems; Cat#5-001-A) was used as an isotype control.

### Phosphatase treatment*, in vitro* binding, immunoprecipitation, western blot, far western and biotinylation assay

λ-Phosphatase treatment,^19^ GST pull down,^19^ far western,^19^ protein extracts preparation,^12^ immunoprecipitation,^12^ immunoblotting assays^12^ and biotinylation assay^12, 62^ were performed as previously described. Immunoblot analysis was performed using the following antibodies: anti-Flag (Sigma, Cat#F3165), anti-Flag-HRP (Sigma; Cat#A8592), anti-MPM2 (05-368, Millipore), anti-Notch1_Val1744_ (Cell Signaling, Danvers, MA, USA, Cat#2421), anti-Notch3 (Cell Signaling; Cat#2889); anti-Notch3 M20 (Santa Cruz Biotechnology, Dallas, TX, USA, Cat# sc-7424), anti-Pin1 (Santa Cruz Biotechnology; Cat#sc-46660), anti-β-actin (Santa Cruz Biotechnology; Cat#sc-47778), anti-Lck (Santa Cruz Biotechnology; Cat#sc-433), anti-α-tubulin (Santa Cruz Biotechnology; Cat#sc-803), anti-LaminB M20 (Santa Cruz Biotechnology; Cat#sc-6217) and anti-Hes1 (Santa Cruz Biotechnology; Cat#sc-25392). The antibody against the activated Notch3-IC protein (N3_IC-act_) was kindly provided by Genentech (South San Francisco, CA, USA). The anti-N3_EC_ (5E1) antibody was kindly provided by Professor A Joutel.^62^ The anti-pTα antibody was kindly provided by H von Bohemer.^63^

### Matrigel invasivity assay

To test the invasion ability, we used Matrigel Invasion Chamber (Corning, Tewksbury, MA, USA; Cat# 354480), as described elsewhere.^31^ Briefly, 18–24 h before transfection (for siRNA experiments), we plate TALL-1 cells at exponential phase in medium 10% FCS plus antibiotics, as recommended by the Neon Trasfection System used for Pin1 silencing. The day after, we silenced Pin1 for 72 h following the Neon manufacturer's instructions and 12 h before the harvesting of the cells, we prepared the Matrigel Invasion Chamber according to the manufacturer's istructions; then, after cell counting we loaded 2.5 × 10^5^ cells into each column (at a final concentration of 0.5 × 10^6^/ml) in medium with low serum. All samples were loaded in triplicate. After 12 h in the incubator at 37 °C, we harvested the cells by recovering the Invasive cells, I (which were passes throught the matrigel until the complete medium) and the Non-Invasive cells, NI (which were retained in the upper space of the chamber). After counting, we proceeded with two type of analysis, obtaining the same results: (a) % invasiveness=[I/total cell count (I+NI)] × 100; (b) % invasiveness=(I/NI) × 100.

For invasion experiments on CD4^+^CD8^+^ DP splenocytes sorted from N3_IC_-tg and N3_IC_-tg/Pin1^−/−^ mice, we proceeded with the same protocol described above after cell sorting; 24 h into Matrigel columns before harvesting was considered.

### Subcellular fractionations

Cytoplasmic-membrane and nuclear-cytosol fractionations were performed as described elsewhere.^12, 64^

### Histological analysis

Non-lymphoid organs were formalin fixed and paraffin embedded. Consecutive sections (5–6 μm thick) were stained with Hematoxylin and Eosin and observed under light microscopy. The analysis was conducted blindly.

### Cell transfections and plasmids

Transient transfections were performed by Lipofectamine-2000 Kit (Invitrogen, Carlsbad, CA, USA), according to the manufacturer's instructions. Expression plasmids used: HA-Pin1 and GST–Pin1,^19^ Flag-N3_IC_ wt^12^ were previously described.

### RT–PCR/qRT–PCR

Total RNA extraction and reverse transcription PCR (RT–PCR) were previously described.^55^ The expression levels of MMP9 and GAPDH mRNA were determined by TaqMan quantitative real-time RT–PCR (qRT–PCR) performed on cDNA according to the manufacturer's instructions (Applied Biosystems, Life Technologies Brand, Carlsbad, CA, USA) and using the ABI Prism 7900HT (Applied Biosystems). Data were analyzed by the ΔΔCt method, and GAPDH was used to normalize the expression levels of mRNA.

### siRNA silencing

Cells were transfected with 20 nM siRNAs anti-Notch3 (Santa Cruz Biotechnology; Cat#sc-37135) or Pin1 (Santa Cruz Biotechnology; Cat#sc-36230) and the corresponding control scrambled siRNAs (Santa Cruz Biotechnology; Cat#sc-37007) using Neon transfection System (Life Technologies; Invitrogen) following the manufacturer's recommendations. Cells were analyzed 48 or 72 h after transfection.

### *In silico* analysis of patient and T-ALL cell lines deposited data

Bone marrow or PB samples from a cohort of 117 pediatric T-ALL patient, deriving from different molecular cytogenetic T-ALL subgroups samples,^26^ and a group of 41 human T-ALL cell lines from three Oncomine datasets^23, 24, 25^ were analyzed for the correlation between Pin1 and Notch3. The expression values of Notch3 and Pin1 were filtered in each analysis utilizing the expression probe set 203237_s_at representing Notch3 and the expression probe set 202927_s_at representing Pin1. The expression value of Notch3 and Pin1 is given in log2 scale after normalizing data with the RMA algorithm.^26^ The index Pearson *R* expresses the linear relation between paired samples, and *P*-values were calculated using Student's *T*-test.

### Fluorescence-activated cell sorting analysis

Freshly isolated cells from thymi, SPL, blood and lymph nodes were prepared and stained, as previously described,^65^ and analyzed on an FACS-Calibur with CellQuest software (BD-Biosciences, San Jose, CA, USA). Cells were stained with APC-CD4 and PE-CD8-conjugated mAbs (BD-PharMingen, San Diego, CA, USA). For Notch3 extracellular staining, cells were incubated with murine Notch3 antibody (R&D Systems; Cat#AF1308) or normal goat IgG (R&D Systems; Cat#AB-108-C) used as a negative control. Cell sorting of Total RNAD4^+^CD8^+^ DP splenocytes suspensions from N3_IC_-tg and N3_IC_-tg/Pin1^−/−^ mice was obtained as described elsewhere.^55^

### Statistical analysis

Results were reported as the mean±s.d. A Student's *t-*test for paired samples was used to assess differences among groups. For multiple comparisons of groups, one-way analysis of variance was used. Among the groups that we have statistically compared we observed similar variance. A P-value of ⩽0.05 was considered as statistically significant (*P⩽0.05; **P⩽0.01 and ***P⩽0.001). In some cases, the index Pearson *R* is also indicated to express a possible linear relation between paired samples. All data shown are representative of at least three independent experiments, and the repeat number was increased according to effect size or sample variation. We estimate the sample size considering the variation and mean of the samples. No statistical method was used to predetermine sample size. No animals or samples were excluded from any analysis.
