# Supplementary material for: Prolyl-isomerase Pin1 controls Notch3 protein expression and regulates T-ALL progression
Source: Oncogene. 2016 Feb 15;35(36):4741–51. doi: 10.1038/onc.2016.5 (PMC5024153; doi:10.1038/onc.2016.5)
Supplement: Supplementary Information [file onc20165x1.docx]

**Supplementary Figures Legends**

**Supplementary Figure S1. Notch3 and Pin1 levels correlate in T-ALLs.**

Graphs showing correlation between Pin1 and Notch3 gene expression levels obtained by an *in silico* analysis using the expression of probes set 203237_s_at and set 202927_s_at representing the Notch3 and Pin1, respectively, in (a) human T-ALL cell lines and (b) a cohort of 117 pediatric T-ALL patients.

In both graphs (a) and (b), each dot corresponds to one patient and the expression value of Notch3 and Pin1 is given in log2 scale after normalizing data with justRMA algorithm normalization. The X-Y axis represent Pin1 and Notch3 expression levels, respectively. The index Pearson R indicated express the linear relation between paired samples and P-values were calculated using Student’s T-test, as described in Material and Methods Section.

**Supplementary Figure S2. Pin1 silencing influences the TALL-1 cells invasiveness by regulating N3_IC_ protein expression.**

Relative cell count after (a) Pin1 silencing and (b) Pin1 silencing plus Notch3 blocking analysed as fold of activation with respect to the negative control, siCTR (a) or siPin1 plus FCNotch3 (-) (b). In all panels, results are shown as the means average deviations of three separate experiments and P-values were calculated using Student’s T-test (i.e., ns, not significant P>0,05).

siPin1: Pin1 silencing; FCNotch3: Notch3 blocking Antibody.

**Supplementary Figure S3. Graphs of DP cells (%) in spleen, peripheral blood and lymphonode of all different mice used in the experiments.**

(a,b) Left panels, graphs of DP cells percentage in (a) spleens and (b) peripheral blood of 6 weeks-old Pin1^+/+^ (n=15), N3IC-tg (n=25) and N3IC-tg/Pin1^-/-^ mice (n=15), whose representative experiment was shown in the Figure 3c. Right panels showed the mean average values and the statistical analysis associated. (c-e) Left panels, graphs of DP cells percentage in (c) spleens, (d) lymphonode and (e) peripheral blood of 10 weeks-old Pin1^+/+^ (n=9), N3IC-tg (n=15) and N3IC-tg/Pin1^-/-^ mice (n=12), whose representative experiment was shown in the Figure 4a. Right panels showed the mean average values and the statistical analysis associated.

P-values were calculated using Student’s T-test (i.e., ns, not significant P>0,05; ***P≤0.001).

**Supplementary Figure S4. Pin1 affects Notch3 processing.**

Optical densitometry (OD) of (a) N3EC in membrane fractions, (b) N3IC in total fraction, (c) N3IC in nuclear fractions, from Pin1^+/+^ and Pin1^-/-^ thymocytes showed in the Figures 6e, 6f and 6g, respectively.

**Supplementary Figure S5. Pin1 ablation induces the increase of N3_EC_ expression per cell in both DP Thymocytes and DP Splenocytes of young N3_IC_ transgenic mice.**

Relative Notch3 extracellular expression (N3_EC_) from gated DP Thymocytes (a) and (b) DP Splenocytes derived from 6-week-old Pin1^+/+^ (A), N3_IC_-tg (B, E) and N3_IC_-tg/Pin1^-/-^ (C, F) mice described in the Figures 3a and 3c, respectively. In both panels (a) and (b), the mean fluorescence intensity (MFI) is also indicated.

Results are shown as the means average deviations of five separate experiments (n=3-5 mice per group) and P-values were calculated using Student’s T-test (i.e., * P≤0.05; **P≤0.01).

**Supplementary Table S1**

**T-ALL cell lines used for *in silico* analysis of Pin1 and Notch3 gene expression levels.**

Table shows the T-ALL cell lines derived from Oncomine datasets, reported in Material and Methods section (Barretina CellLine, Garnett CellLine and Palomero CellLine), used for *in silico* analysis shown in the Supplementary Figure 1a.
