# Supplementary figures and images for: Prolyl-isomerase Pin1 controls Notch3 protein expression and regulates T-ALL progression
Source: Oncogene. 2016 Feb 15;35(36):4741–51. doi: 10.1038/onc.2016.5 (PMC5024153; doi:10.1038/onc.2016.5)

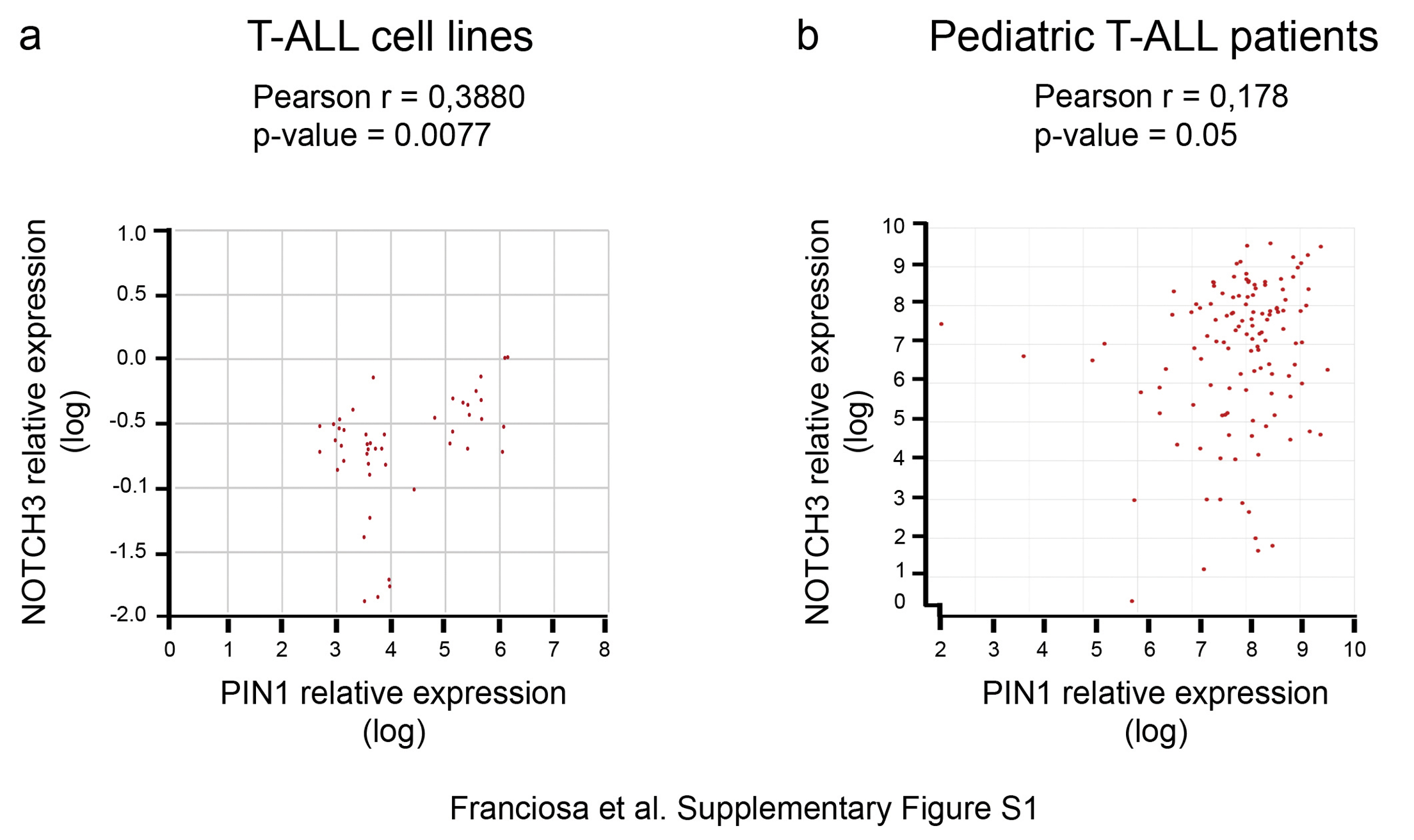

Supplement: Supplementary Figure S1 [file onc20165x2.tif]

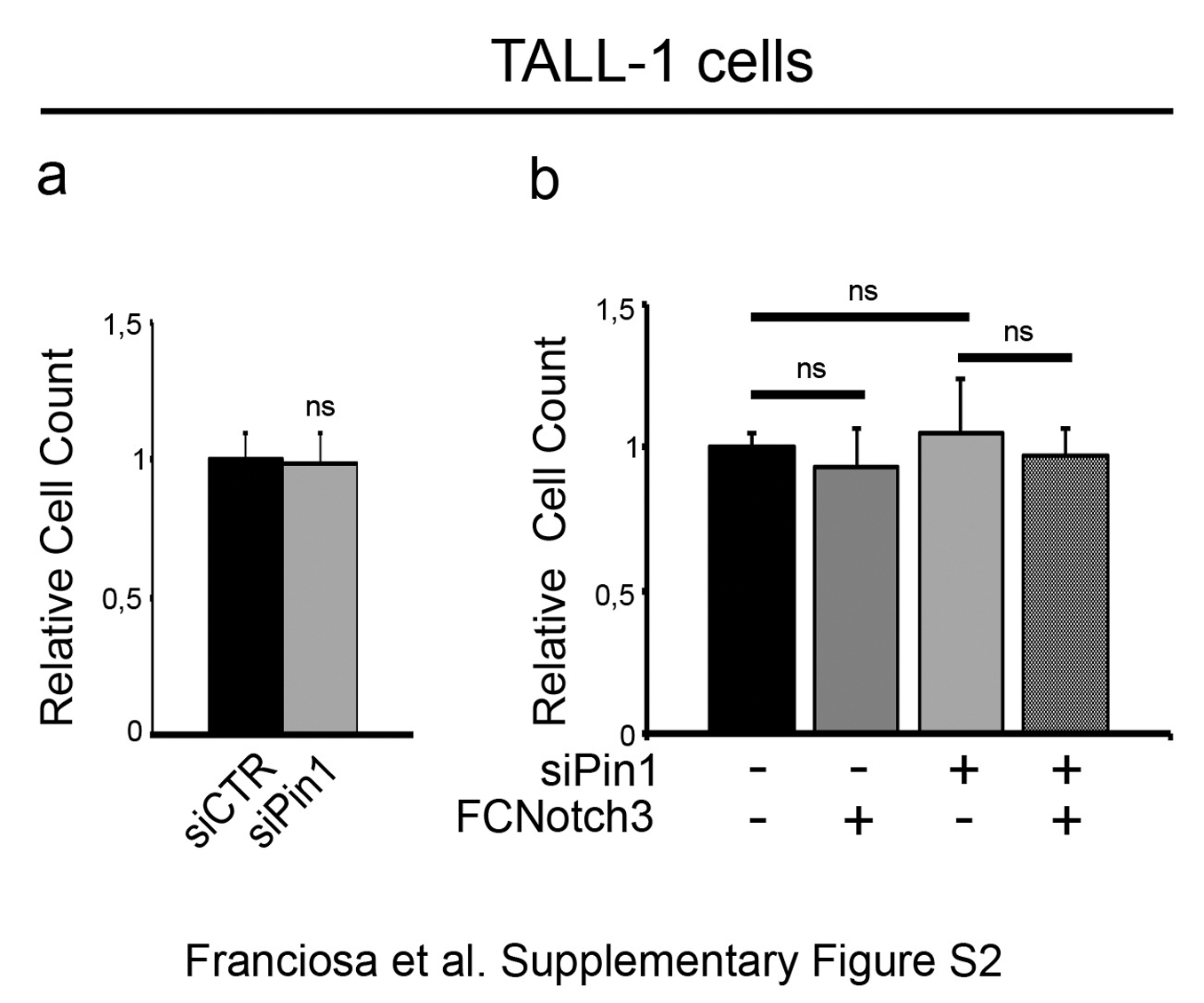

Supplement: Supplementary Figure S2 [file onc20165x3.tif]

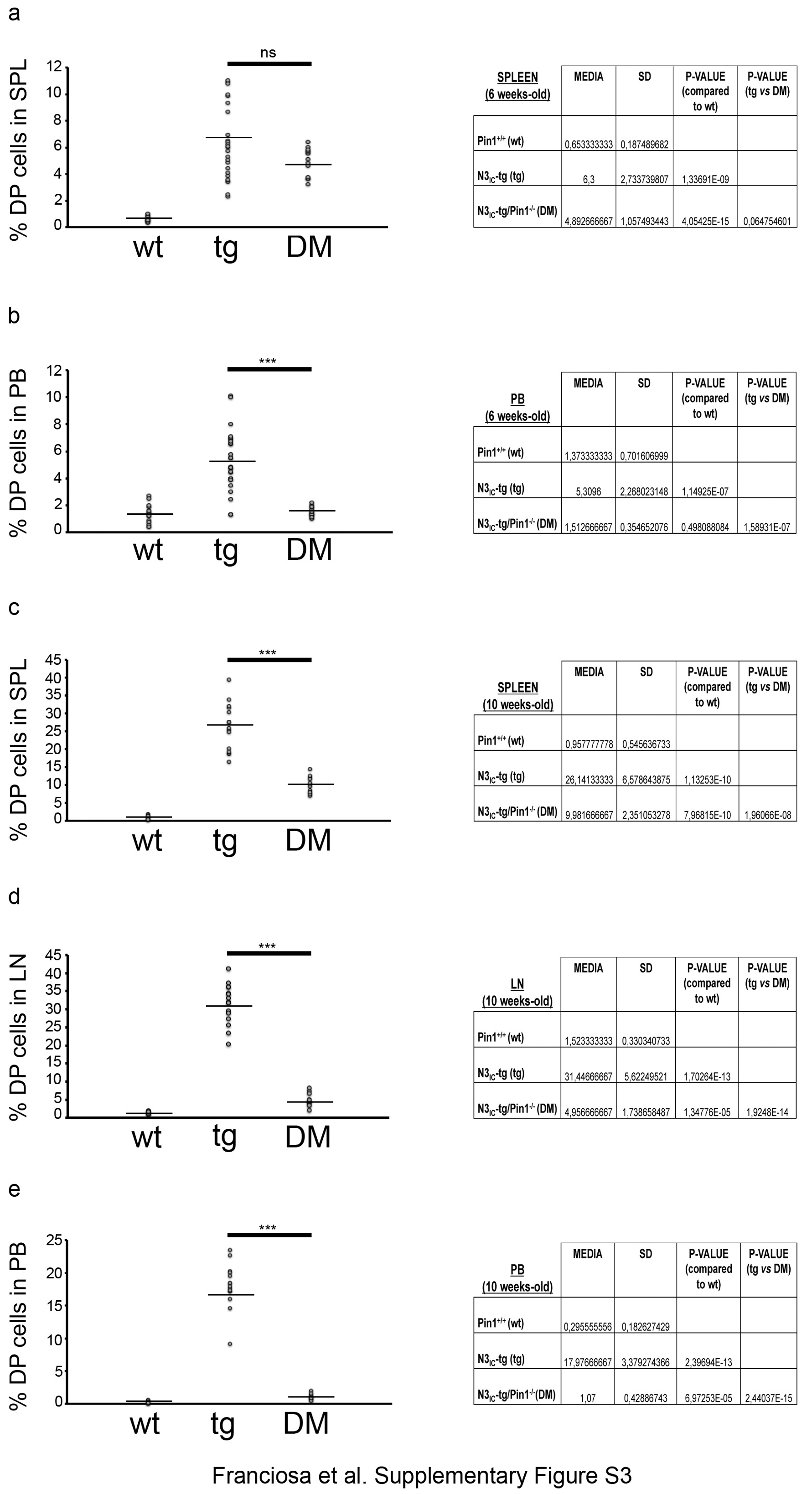

Supplement: Supplementary Figure S3 [file onc20165x4.tif]

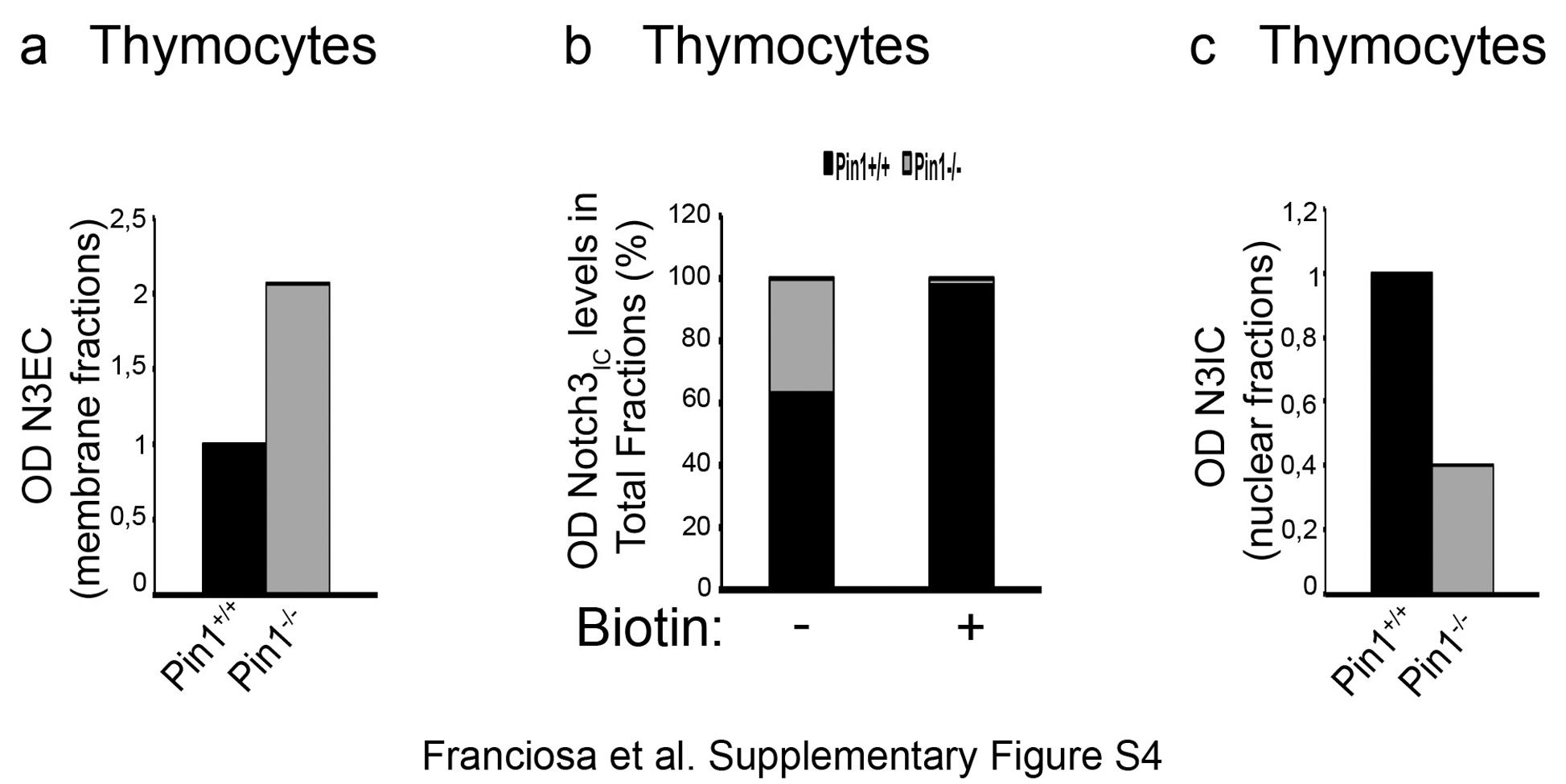

Supplement: Supplementary Figure S4 [file onc20165x5.tif]

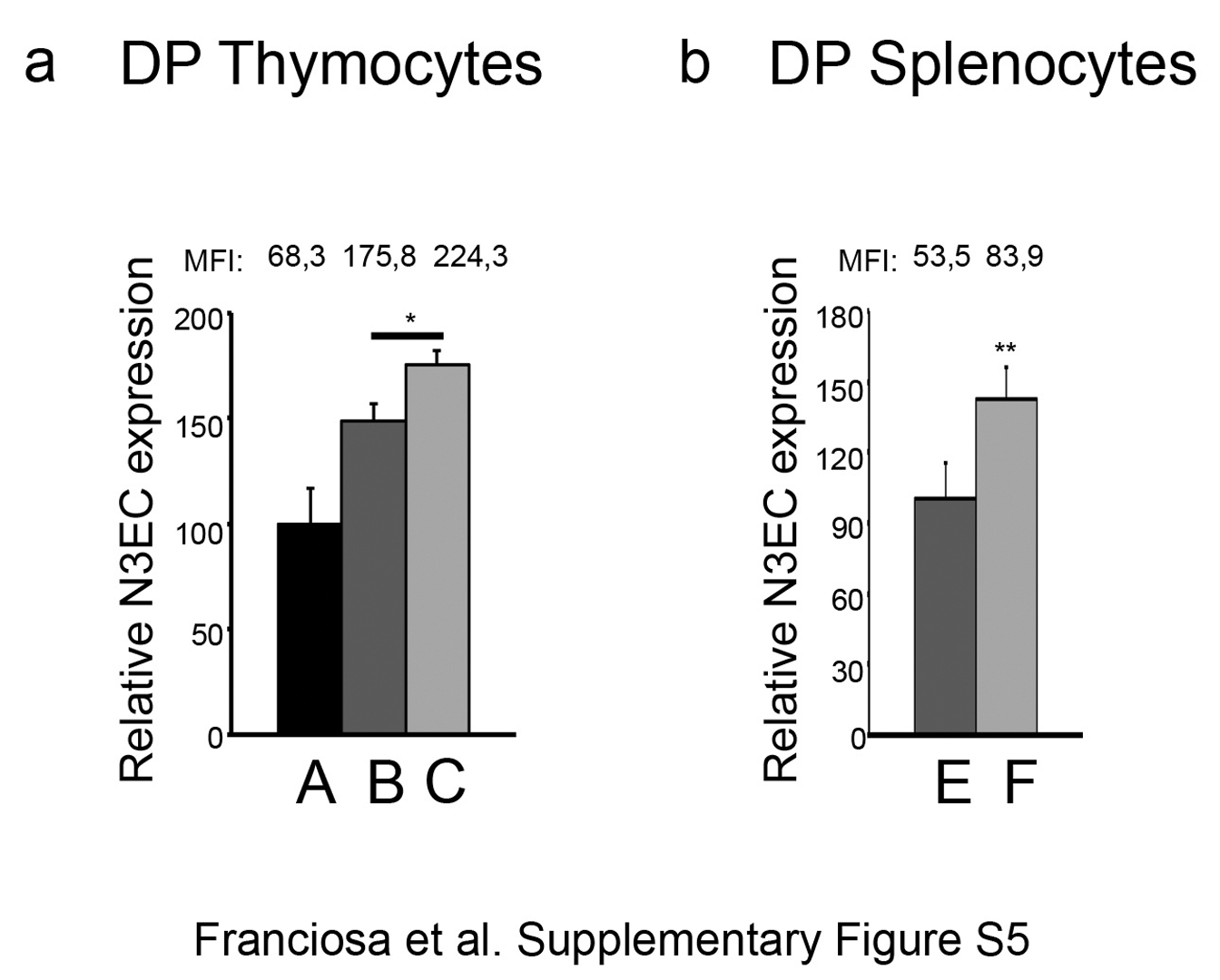

Supplement: Supplementary Figure S5 [file onc20165x6.tif]

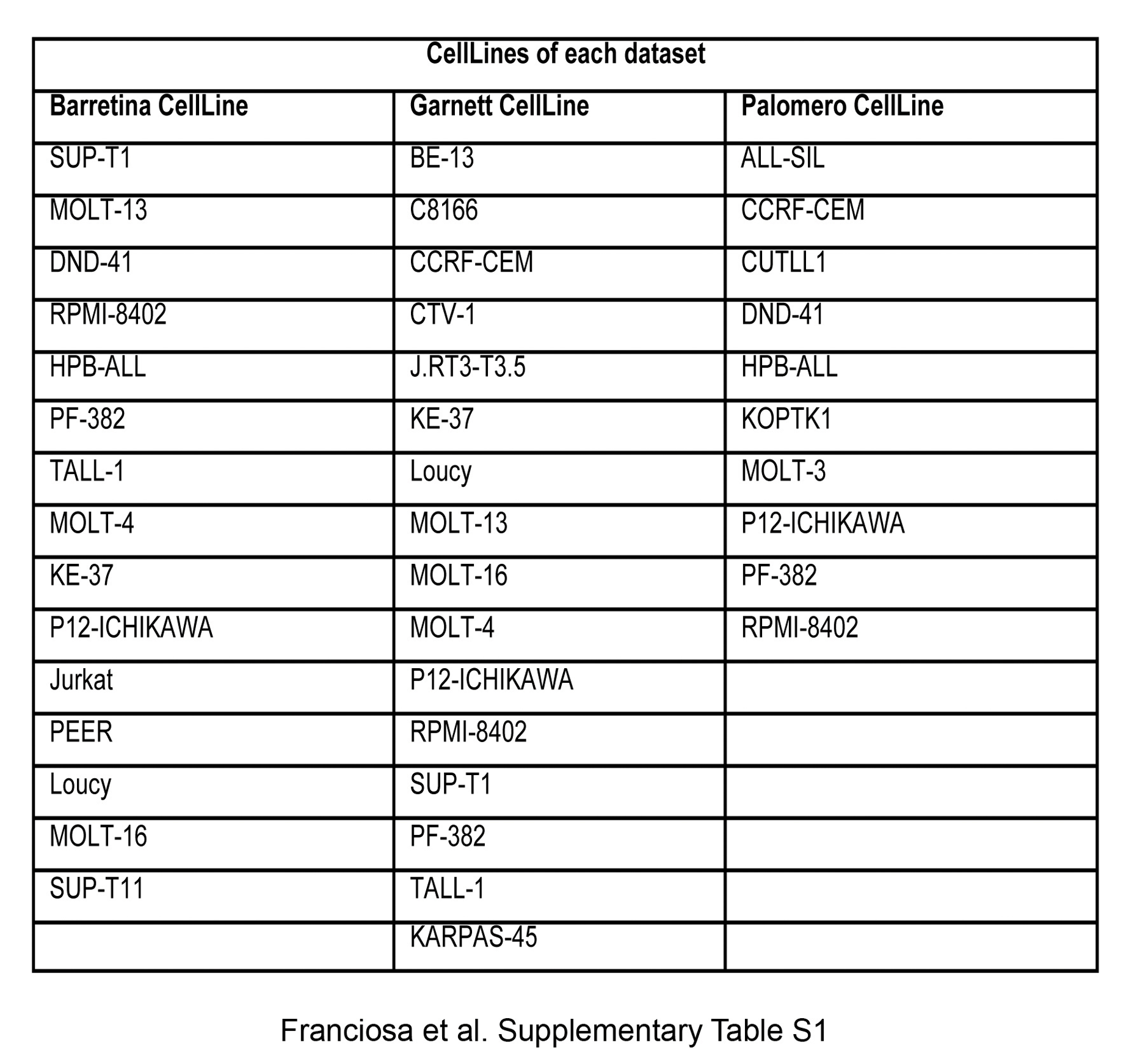

Supplement: Supplementary Table S1 [file onc20165x7.tif]
